# Use of Artificial Intelligence to Identify New Mechanisms and Approaches to Therapy of Bone Disorders Associated With Chronic Kidney Disease

**DOI:** 10.3389/fmed.2022.807994

**Published:** 2022-03-25

**Authors:** Adam E. Gaweda, Eleanor D. Lederer, Michael E. Brier

**Affiliations:** ^1^Division of Nephrology, Department of Medicine, University of Louisville School of Medicine, Louisville, KY, United States; ^2^Medical Services, VA North Texas Health Sciences Center, Dallas, TX, United States; ^3^Division of Nephrology, Department of Medicine, University of Texas Southwestern Medical Center, Dallas, TX, United States; ^4^Charles and Jane Pak Center for Mineral Metabolism and Clinical Research, University of Texas Southwestern Medical Center, Dallas, TX, United States; ^5^Research Service, Robley Rex VA Medical Center, Louisville, KY, United States

**Keywords:** osteoporosis, chronic kidney disease, artificial intelligence, mathematical modeling, *in silico* clinical trials

## Abstract

Chronic kidney disease (CKD) leads to clinically severe bone loss, resulting from the deranged mineral metabolism that accompanies CKD. Each individual patient presents a unique combination of risk factors, pathologies, and complications of bone disease. The complexity of the disorder coupled with our incomplete understanding of the pathophysiology has significantly hampered the ability of nephrologists to prevent fractures, a leading comorbidity of CKD. Much has been learned from animal models; however, we propose in this review that application of multiple techniques of mathematical modeling and artificial intelligence can accelerate our ability to develop relevant and impactful clinical trials and can lead to better understanding of the osteoporosis of CKD. We highlight the foundational work that informed our current model development and discuss the potential applications of our approach combining principles of quantitative systems pharmacology, model predictive control, and reinforcement learning to deliver individualized precision medical therapy of this highly complex disorder.

## Introduction

The bone abnormalities associated with chronic kidney disease, so-called renal osteodystrophy, are part of a clinical syndrome called chronic kidney disease-mineral bone disorder (CKD-MBD) that includes renal osteodystrophy, abnormalities of mineral metabolism, and vascular calcification. Osteoporosis, defined by the National Osteoporosis Foundation (now called the Bone Health and Osteoporosis Foundation) as a decrease in bone mineral associated with loss of bone architecture and strength (1), may be a contributory factor for renal osteodystrophy, but is only one of many different pathologies that affect the bone health of patients with CKD (2). The management of renal osteodystrophy presents significant challenges due to the broad heterogeneity of contributing factors, each of which may have differing impacts in individual patients. Chief among these factors are derangements in the regulation of vitamin D, parathyroid hormone (PTH), calcium homeostasis, phosphate homeostasis, acid base balance, sex hormones, and a variety of more recently discovered mediators including sclerostin, fibroblast growth factor 23, dickkopf 1, activin A, and numerous inflammatory agents. In the absence of a bone biopsy, it is not possible to definitively diagnose the specific bone disease present in an individual patient; therefore, throughout this article, we will use the term CKD-MBD instead of osteoporosis. The goal in addressing this complex disorder is to develop specific treatments based on the identification of modifiable alterations in the risk factor profile for each patient, allowing for an individualized precision approach, to prevent fractures and accompanying soft tissue calcification. In many ways, this approach has been realized in oncology, but little progress in this realm has been made in nephrology (3, 4).

Two recent developments suggest that the treatment of CKD-MBD is a promising clinical area to apply the principles of personalized medicine. The first is precision drug dosing to achieve pharmacologic targets within a narrow therapeutic range. This therapeutic avenue originates within the engineering literature and centers on the application of artificial intelligence, machine learning, and control theory to administer pharmacologic agents precisely in the dialysis patient population (5, 6). A second advancement is the development of a quantitative systems biology model of osteoporosis. In science, we often develop models of human disease in animals in which we can rigorously test the impact of manipulations of specific biochemical processes to observe their outcomes. We then develop and test hypotheses in human beings based on extrapolation of the results in animal models using the clinical trial mechanism, ultimately resulting in therapeutic guidelines. We propose that the future direction in the treatment of CKD-MBD in chronic kidney disease can be mapped out through merging a systems biology model with state-of-the-art engineering techniques. This approach can identify and test therapeutic manipulations to determine the effects on disease parameters that are routinely measured, those that are not routinely measured, and even parameters that at this time are clinically unmeasurable but ultimately result in relevant clinical outcomes such as bone fracture. In this manuscript, we will discuss the steps toward achieving these clinical goals through the application of a systems biology approach to this complex clinical disorder.

## Development of a Mathematical Model of Chronic Kidney Disease-Mineral Bone Disorder

The first step in the development of a tool for the precision dosing of drugs used to treat CKD-MBD is the development of a model system. The traditional approach would be the development of a murine model of CKD-MBD (7). For example, dilute brown non-Agouti (DBA/2) mice, when fed a high phosphate diet, are susceptible to develop medial vascular calcification and low turnover bone disease, a reasonable animal model to test therapeutic strategies for the treatment of vascular calcification and renal osteodystrophy. Animal models, however, present significant limitations. First, decades of experience with mouse models have demonstrated the pitfalls in extrapolating information learned in the mouse to human pathophysiology. Second, multiple different animal models are required to investigate the varied abnormalities found in CKD-MBD. Not all patients with CKD and renal osteodystrophy have low turnover bone disease and even within the category of low turnover bone disease, not all CKD patients demonstrate the same pathophysiology (8, 9). One additional issue is that these studies are generally performed in an animal strain, thus limiting the ability to assess inter-animal variability in response to therapies. Mice show significant genetic diversity in bone disease, even in the absence of CKD. Third, assessing the effect of multiple interventions can require very large numbers of animals and the development of multiple protocols resulting in considerable time and financial investment.

An alternative tool to investigate CKD – MBD is a comprehensive mathematical model of a human, developed using information from animal studies and from measurements of key biochemical components in humans. In the parlance of the contemporary literature, this approach is referred to as a quantitative systems pharmacology (QSP) modeling. A QSP model is robust and can be easily altered as new information is acquired. Biochemical processes can be described by ordinary differential equations, good examples being the mathematical properties of enzyme kinetics which often follow a Michaelis-Menten process. Using a library of mathematical functions, we can describe the concentration of a substance in the body over time and how that substance can interact with any biological process. A combination of these equations can then be used to describe the activity of a specific enzyme, a combination of enzymes and systems can describe an organ, and by linking the effects on different organs, we attempt to describe the human.

**FIGURE 1:**
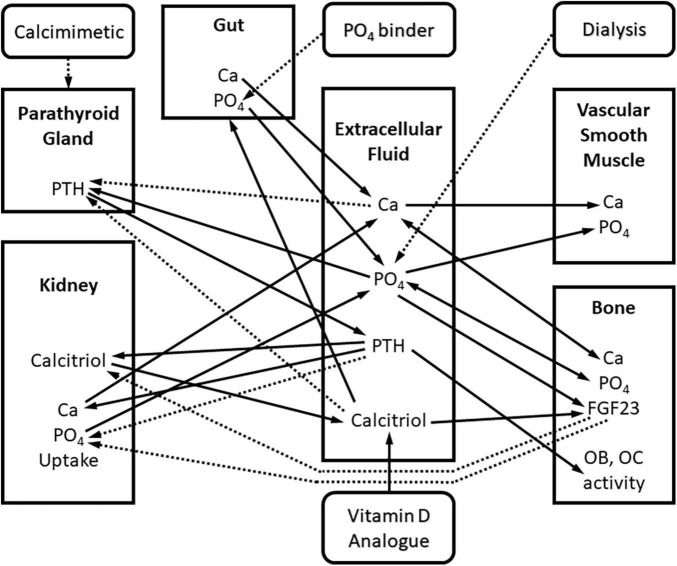
Block diagram of the quantitative systems pharmacology model of Ca and PO_4_ metabolism in chronic kidney disease. Square blocks represent compartments, rounded blocks represent interventions. Arrows represent interactions between biomarkers and compartments: continuous – positive interaction, dashed – negative interaction. Interactions are mathematically described using ordinary differential equations (ODE’s).

We have published an example of a QSP model of CKD-MBD that spans all stages of CKD and incorporates kidney replacement therapy, and the three pharmaceutical agents commonly used in the treatment of advanced CKD-MBD (10). This model is shown in Figure 1 as several interconnected compartments, including kidney, bone, soft tissue, and parathyroid gland. This model was derived from the published literature (11–13) and validated using human data collected in the Chronic Renal Insufficiency Cohort (CRIC) study (14, 15). Using this QSP model, we were able to describe the time course of the changes in serum concentrations of calcium (Ca), phosphorus (P), and parathyroid hormone (PTH), the three parameters routinely measured as part of clinical practice in the treatment of CKD-MBD as recommended by the Kidney Disease Improving Global Outcomes (KDIGO) guidelines (16). Additionally, the changes in the concentration of biochemical parameters such as FGF23 and calcitriol, that can be, but are not routinely measured clinically, were accurately predicted. Ultimately, the main strength of this model lies in the ability to predict unmeasured and practically unmeasurable parameters such as flux of calcium and phosphate from bone to serum and from serum to soft tissue, the biologic processes that directly relate to and predict the morbidity associated with CKD-MBD like renal osteodystrophy and vascular calcification.

One immediate application of this model would be to study the effect of pharmacologic manipulation of the model to investigate treatment alternatives. Potentially informative queries would be: (1) Of the three, Ca, P, PTH, which should be prioritized to minimize calcium efflux from the bone and influx into soft tissue? (2) Given the limitation of the maximum tolerated dose of phosphate binders which agent, calcimimetic, or calcitriol should be prioritized? (3) What serum concentrations of Ca, P, and PTH should be targeted to minimize calcium efflux from the bone and influx into soft tissue? To give a very simple example, through the use of simulated patient data, the investigator could ask the model to predict mineral flux with the use of only calcimimetics and phosphate binders vs the use of only calcitriol and phosphate binders. Based on the results, a hypothesis would be formulated and tested in a population of patients with CKD. Multiple questions of increasing complexity, reflecting the complexity of the clinical disorder, could be posed to the model and the most promising avenues subsequently tested in human studies, potentially saving time and money.

Decisive advantages in working with a QSP model is the flexibility to update the model as new information is obtained and to query the model to determine which parameters to manipulate to optimize response. Newer therapies such as antibodies against sclerostin or FGF23 can be incorporated into the model. Additional markers and mediators of CKD-MBD such as sex hormone concentration, urine citrate, and inflammatory cytokines can be included. The model can be aged *in silico* in several ways such as decreasing the glomerular filtration rate and thereby decreasing phosphate excretion. Also, those factors that change with age or even sex can be coded into the mathematical relationships within the model which can subsequently be tested against any number of hypotheses.

## Introduction of Artificial Intelligence

Teaching a computer to “think” like a human would has been attempted for over half a century. Turing first introduced the idea in 1950 but only recently have applications of artificial intelligence penetrated into medicine (17). The ostensibly simplistic goal of Turing’s experiments was to see if a human subject could discriminate between decisions made by a computer that had been trained to think like a human and decisions made by a human. Ideally, if the computer was adequately trained, the human subject would not be able to make this distinction. In medical literature, Artificial Intelligence is being touted as an attractive methodology to deal with extraordinary amounts of data (Big Data) that cannot be easily analyzed by the human brain. That is, as we combine information from the electronic medical records with -omic data and data from other sources, we need a unifying processing framework which we can utilize these data to benefit the patients.

Present day Artificial Intelligence methods are founded on two algorithms that we can use to “teach the computer to think”: Supervised and Unsupervised Learning. In Supervised Learning, the training data that the computer uses to decide is paired with a predetermined, presumably correct, response. The computer learns to associates input patterns with a particular outcome. The most common tool used in this approach is the Artificial Neural Network (ANN). Artificial Neural Networks are built upon a machine construct of a human neuron, where dendrites collect input and compute a response in the neuron cell body and pass this information to the next neuron through the axon. An ANN is a collection of neurons, mathematically represented as a set of complex non-linear equations. A standard ANN typically uses a single processing layer (hidden layer) of neurons that stores information estimating the importance of each input and the associations between inputs and outputs. Deep Neural Networks (DNN) are an extension of ANN’s which add multiple hidden layers of neurons with different processing functions. DNNs have gained popularity due to their success with high dimensional data problems, such as image and text classification (18).

The main goal of Supervised Learning is to efficiently replicate the medical expertise developed through clinical experience and encoded in the training data. Certainly, there is a role for the development of this type of application within the medical realm. A good example is the interpretation of kidney biopsy material (19, 20). Using this application, investigators reviewed 2,542 kidney biopsies and 12,259 immunofluorescence images. The accuracy of the artificial intelligence approach to the tissue diagnosis ranged from 79 to 94% compared to the pathologist diagnosis. The authors considered this level of concordance comparable to the performance by a human pathologist. The major gain derived from the AI approach was the fact that the images were processed 117 times faster; however, there was no increase in accuracy. The authors acknowledge that the results may have been influenced by the single center nature of the obtained sample and that the process may not identify rare diagnoses since those cases may not be available for “training” the neural network. This manuscript illustrates a common misconception about the capabilities of artificial intelligence. A supervised learning approach will interpolate well within the range of the data with which it is trained but cannot extrapolate outside of that data. The expectation that a supervised artificial intelligence approach will yield superior outcomes in terms of accuracy of diagnosis or novel clinical insights is a fallacy. These models must be rigorously tested during their development and then validated against a separately derived data set to ensure reproducibility which does not always occur. Further, these methods need to be tested against standard statistical tools to show superiority rather than novelty.

In Unsupervised Learning, the Artificial Intelligence agent independently organizes data into similar groups. Using the example above of interpreting biopsy images, an Unsupervised Learning could be used to group image data based on pixel intensity (image segmentation) and look for correlations in those groups when compared to the pathology report. One would then need to investigate the features that are being sorted like cellularity within the glomerulus, thickness of the basement membranes, presence of a crescent, or other pathologic parameters. This is like the statistical technique called factor analysis or nearest neighbor. Using this methodology, the incorporation of Artificial Intelligence has the capability of identifying new or previously unrecognized patterns and thus could suggest new classification schema (21–24). Thus, Unsupervised Learning holds the promise of introducing new ideas and generating new hypotheses of disease development or progression.

A methodology that combines features of Supervised and Unsupervised Learning is Random Forest (25). A Random Forest (RF) approach looks to discover relationships between sets of data that will allow a decision tree to be produced that will allow categorization of patients. Using this application, the authors of a recent study leveraged the availability of large data sets and machine learning approaches to generate new information about CKD and parameters of mineral metabolism. The analysis allowed the authors to discover strong associations between PTH and phosphate, clarifying how phosphate modifies PTH in association with calcium and other variables. The authors were able to demonstrate a stronger association between phosphate and PTH than using a traditional linear regression analysis. They could also evaluate the effect of pharmacologic intervention in these patients.

## From Model Development to *in silico* Testing in Chronic Kidney Disease-Mineral Bone Disorder

Several models for the approach to kidney disease have been discussed beginning with animal models which then lay the framework for the mathematical models that we have described: the QSP model, the QSP model incorporating supervised learning, and the model incorporating unsupervised learning. Any of these models can be used to investigate human disease to varying levels of success. Animal models allow for the investigation of specific genetic manipulations in the whole animal. Mathematical models can help achieve the same goal. However, the speed and breadth of the testing that can occur in a mathematical model is far beyond what can be done in individual animal models.

To that end, we can use the CKD-MBD model to perform *in silico* testing. In this process, we use the computing power to run thousands or millions of iterative simulations to evaluate the impact of time, disease, and pharmacologic manipulation on the model. Such models have been developed and tested over the last decade, two of which are particularly pertinent to the subject of renal osteodystrophy. In one published example, the authors developed a model of bone biology and calcium homeostasis using literature-derived data on the relevant specific parameters, including serum PTH, calcitriol, calcium and phosphorus (7–9). They used this model to perform *in silico* studies of the effect of hyper- and hypoparathyroidism, kidney disease, and the pharmacologic agents PTH 1-34 and denosumab, the receptor activator of NF-kappaB ligand (RANKL) inhibitor (9). In their first work, the authors used the model to link bone remodeling markers with bone mineral density. They also simulated the effect of administration of a calcimimetic and calcitriol on the metabolic processes represented by the model. Subsequently, the authors describe the administration of the monoclonal antibody denosumab used in the treatment of osteoporosis. In the same publication, the authors demonstrate the robust ability of the model to incorporate new information in order to make predictions with a new therapeutic agent. The authors conclude that their model can be used as a platform for evaluating therapeutics. Another published example described the regulation of the parathyroid gland (26), incorporating the major mechanisms of production, secretion, and degradation of PTH. These authors also recognize the utility of the model in evaluating therapeutics.

## Therapy Individualization in Chronic Kidney Disease-Mineral Bone Disorder

The premise of this article is to address frontiers in treatment of CKD-MBD. We have already discussed briefly how both animal and mathematical models are being used to describe the disease process of CKD-MBD. These models have been developed to mimic both individual organs and the whole organism. This information can be used to investigate the effects of new pharmacologic agents both, in living beings and virtually *in silico*. We would like to conclude this overview with advancements using the above referenced work and merging it with the concept of control theory in order to achieve personalized precision therapy of CKD-MBD.

Our group has been at the forefront of the development of novel ways to individualize therapy for patients with chronic kidney disease. Our initial work centered on the management of anemia in ESRD patients. Achieving guidelines goals for anemia management has been challenging in this population in large part due to the practice of addressing the erythropoietin dosing monthly while recognizing that the RBC lifespan is 90 days, creating a disconnect between prescription and outcome assessment. To address this complexity, we introduced an additional AI tool, Model Predictive Control (MPC). This AI application has two requirements: a model of the system of interest and a controller. The model can be mechanistic, as described in a QSP model. Alternatively, the model can be an Artificial Neural Network which, like the RF model discussed above, is applicable only for patients treated in the same manner as the patients whose data were used to develop it (27). The second part of MPC is the controller, which is equipped with information about the goals of the therapy. For our work, an AAN model of erythropoietin therapy for patients with the anemia of CKD was developed incorporating the target hemoglobin, erythropoietin type, maximum dosage, as well as other factors the physician would take into consideration. The controller would then interrogate the model of the patient to determine the optimal erythropoietin dose based on the patient’s prior response to erythropoietin and current hemoglobin level. A decision support tool based on this work is being used in clinical practice to monitor the anemia of chronic kidney disease patients.

We are now actively pursuing expansion of this methodology to include the treatment of CKD-MBD. We have already developed the model of the patient that can be used by a controller to determine the results of some manipulation whether it be age, decrease in renal function, or therapeutic intervention. Although we could apply the MPC approach that has already proved effective in the case of anemia management, due to the complexity of CKD-MBD and the desire to discover new information, we approach this problem using yet another tool out of the Artificial Intelligence toolbox, called Reinforcement Learning (RL). RL is a family of psychology-inspired methods based on the concept of active learning performed by an intelligent Agent to maximize a cumulative reward related to user-specified goal. The RL Agent performs actions in response to observations it makes and constantly adjusts the mapping between observations and actions. For every action it takes, the Agent receives a reward signal which measures how closely the results controlled by Agent’s actions matches the specified goal. When the reward increases, the Agent’s actions are reinforced. Conversely, if the reward decreases, Agent is discouraged from such actions. In this sense, RL is a hybrid between Supervised and Unsupervised Learning. The supervision comes in the form of a pre-specified goal. However, the Agent learns the observation-action mapping on its own. Compared to Supervised and Unsupervised Learning which strictly depend on the training data, RL has the unique ability to discover novel information by interactively generating new data (28).

The current treatment of CKD-MBD often follows a somewhat linear and uniform pattern guided by clinical practice guidelines that specify the serum concentrations of calcium, phosphorus, and PTH that are desired (29). Patients are generally administered a phosphate binder at the initiation of dialysis. Subsequently, a vitamin D drug is added if a phosphate binder alone does not result in the desired KDIGO goals for PTH and calcium. Finally, calcimimetics are added for failure of PTH control or adverse effects on Ca and P levels. The treatment regimen is complicated by the reported poor adherence to phosphate binders and tolerability of the oral calcimimetic. Using a QSP model-based RL, we can virtually design a dosage regimen that maximizes the percentage of calcium, phosphorus, PTH measurements that are within their target ranges. Based on our prior experience in this area (1), we are confident that a new dosing tool could be developed, and we have developed the controller and have completed simulated clinical trials using this model.

Thus far, we have discussed the application of AI to achieve the biochemical guidelines established for the treatment of CKD-MBD, as these simple biochemical parameters – calcium, phosphorus, and PTH – represent the current standard of practice targets. The more significant power of this AI approach is the ability to study the effects of therapeutics on more clinically relevant outcomes. The goal of the treatment of CKD – MBD is to decrease morbidity and mortality in chronic kidney disease. The morbidity and mortality are primarily associated with the movement of calcium from the bone leading to loss of bone mass and structure and a high fracture rate in this patient population and the movement of calcium into tissue leading to cardiovascular complications. Our QSP model not only predicts the concentrations of calcium, phosphorus, and PTH but also predicts the flux of calcium into and out of the bone and soft tissue. Using the RL tool combined with the QSP model we can design a reward structure that prioritizes the administration of a single or multiple therapeutic agents to optimize calcium flux. Given specific safeguards like avoiding low serum calcium which can also be encoded into the reward function, we can propose different calcium, phosphorus, and PTH targets. Further, scenarios where two or three of the agents used to treat CKD – MBD are manipulated simultaneously can be tested.

## An Example of Using a Quantitative Systems Pharmacology Model and Reinforcement Learning

In this example we will combine that model with a RL approach to simulate the change in model parameters in patients with stage 5 CKD on dialysis and how we can examine the measured, unmeasured, and unmeasurable parameters within the model to guide therapy (30). In this case, the reward function is simply the attainment of the KDIGO recommended concentrations of calcium, phosphorus, and PTH. The reward function can take many forms where each of these three concentration objectives can have their own weight; however, in this example each concentration is weighted equally. The reward function can also use the unmeasured or unmeasurable parameters or any combination of parameters.

**FIGURE 2:**
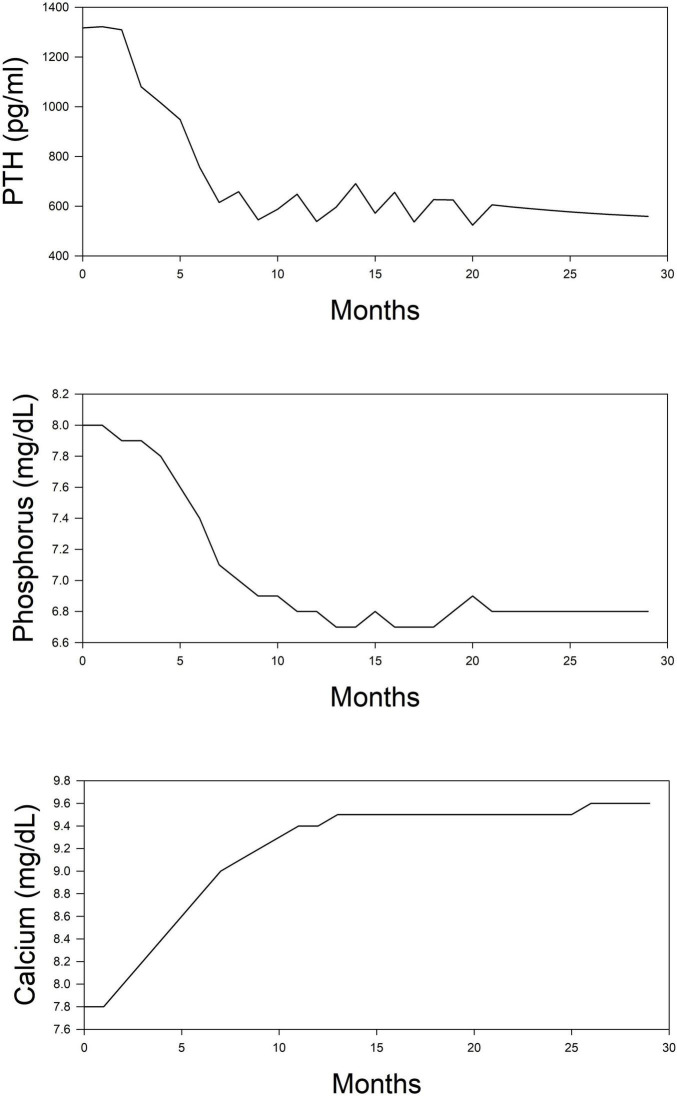
Simplified depiction of the predicted time course of routinely measured markers of mineral metabolism in the simulated (*in silico*) treatment of CKD-MBD using phosphate binder, vitamin D analog, and a calcimimetic. Top panel, PTH; Middle panel, phosphorus; and Bottom panel, calcium.

**FIGURE 3:**
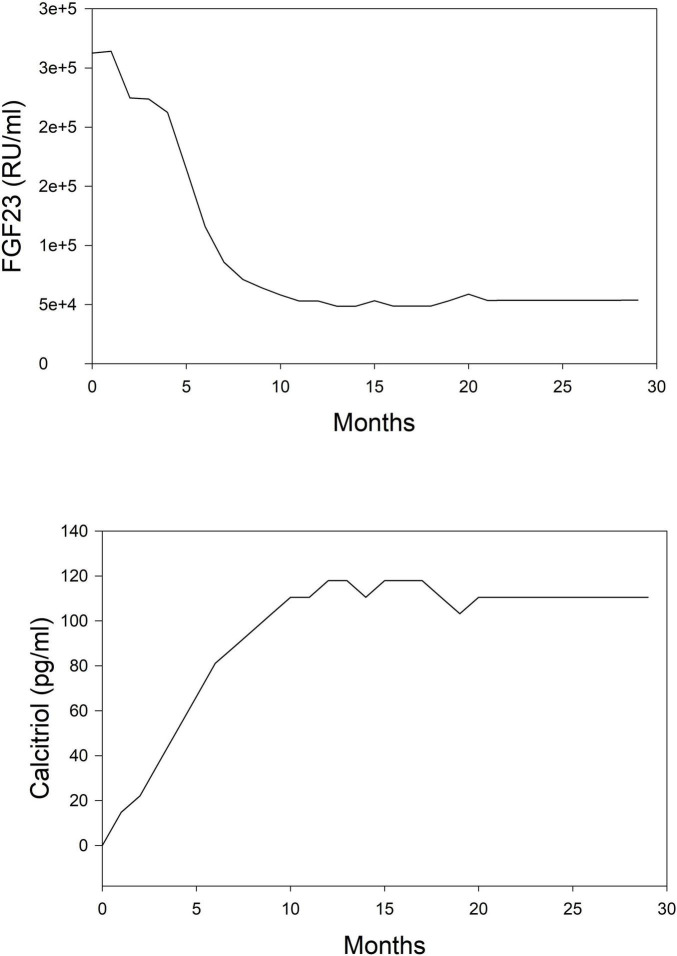
Simplified depiction of the predicted time course of non-routinely measured markers of mineral metabolism in the simulated (*in silico*) treatment of CKD-MBD using phosphate binder, vitamin D analog, and a calcimimetic. Top panel, FGF 23; Bottom panel, calcitriol.

The simulation was performed in the computer program Matlab using the reinforcement learning toolbox. Simulations were run for 30 months following the initiation of dialysis. Starting concentrations of calcium, phosphorus, and PTH were randomly drawn from a range of values normally seen at initiation. Using the reinforcement learning algorithm, the program determines the drug to administer and at what dose. The program does not prioritize one drug over another but purely makes its decision based on a trial-and-error process. The results of this simulation in one subject are shown for illustration purposes. In Figure 2, we can see the model predictions for serum measurements of calcium, phosphorus, PTH which comprise the measured parameters in CKD-MBD. As the computer learns the pharmacodynamics of the system it achieves a lowering of PTH and phosphorus and an increase in calcium concentrations within the first 5 months. In Figure 3, we can see the unmeasured parameters (FGF23 and calcitriol). The model predicts a corresponding drop in FGF 23 concentrations over this time as well as an increase in calcitriol. The increase in calcitriol is a result of the administration of exogenous drug and does not represent increased production.

**FIGURE 4:**
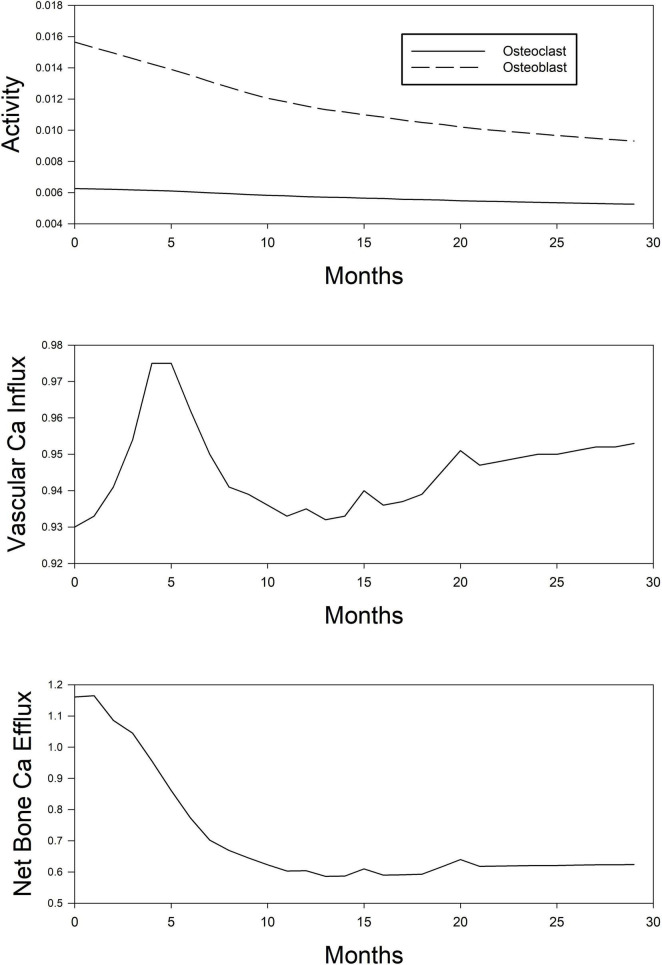
Simplified depiction of the predicted time course of model-estimated (unobservable) parameters of mineral metabolism in the simulated (*in silico*) treatment of CKD-MBD using phosphate binder, vitamin D analog, and a calcimimetic. Top panel, osteoblast (dashed line) and osteoclast (continuous line) activity. Middle panel, Ca influx into the vascular smooth muscle cell tissue. Bottom panel, net Ca efflux out of the bone compartment.

Figure 4 shows the unmeasurable parameters in CKD-MBD (calcium flux, osteoblast and osteoclast activity). Using the KDIGO recommended targets for calcium, phosphorus, and PTH the model would predict that little change occurs in the movement of calcium into tissue, but bone calcium loss decreases by about 50%. Using the approach that we have outlined; one could run different simulations where we optimize calcium movement into the tissue and from the bone to investigate a different dosing strategy.

According to the model, the manipulation of the simulated patient using pharmacologic agents will result in decreased activity in both osteoclasts and osteoblast with the ratio of blast to osteoclasts decreasing over time. Information obtained from the model on these parameters may be useful in determining low and high turnover bone disease or how the administration of newly developed compounds may impact activity.

## Summary

The medical community has shown increasing acceptance of machine learning techniques in the practice of medicine. In this review, we have highlighted several Artificial Intelligence techniques that can be applied to clinical conditions and suggest that this toolbox offers a variety of approaches for understanding pathogenesis of disease, outcomes, and response to therapy. Our success using a machine learning approach in the treatment of anemia of chronic kidney disease suggests that we will be able to produce similar results in CKD-MBD. The advantages and disadvantages of the different models we have discussed are summarized in Table 1. As the complexity of the drug dosing problem escalates such as in the administration of three agents simultaneously the power of these new approaches will increase. New models that specifically investigate the biochemical and cellular processes that occur within the bone that can lead to renal osteodystrophy are needed. Major strengths of the *in silico* AI approach are the flexibility in modifying the model as new clinical findings emerge, the predictive capacity in the context of a multifaceted clinical syndrome, and the ability to generate and test novel hypotheses rapidly and safely. These novel hypotheses, tested *in silico*, can then be translated into more conventional human-based trials, optimized through the preliminary simulations. These models will enable the rapid testing of new therapeutic approaches, including those biochemical processes that may be targeted for the development of new agents to improve care.

**TABLE 1:** Comparison of animal models and machine models of human disease.

Model type	Advantages	Disadvantages
Animal	Biologic model Familiarity within scientific community Ability to constrain subject variability	“Black box” to some extent, not all variables described Applicability to human disease inconsistent Number of replicates and variables limited Lack of generalizability from one well defined animal model to another or from one specific set of experimental constraints to another Need for multiple models, increasing with greater complexity of biologic process Expensive
AI – Supervised learning	Accuracy of output easily compared to MD performance Can be updated for new discoveries in diagnosis and therapies Analysis of clinical data very rapid	Lack of familiarity by clinicians Output defined by current biologic and therapeutic assumptions Inability to identify novel associations or disease processes
AI – Unsupervised learning	Can identify new patterns of clinical disease Can be used for *in silico* trials for hypothesis testing Can be used to predict difficult to measure biologic parameters Can be used for individualized precision medicine Can be updated for new discoveries in diagnosis and therapies	Lack of familiarity by clinicians Reluctance on the part of clinicians to accept predictions that are contrary to prior understanding/practices Accuracy of predictions dependent on accuracy and completeness of model parameters

## Author Contributions

All authors contributed to the concept and design of the manuscript, the initial drafting of different portions of the manuscript, and the revisions resulting in the final submission. MB and AG were responsible for changes in the figures in response to the reviews. EL was responsible for changes in the verbiage in response to the reviews. All authors approved the final form of the manuscript and stand by its contents.

## Conflict of Interest

The authors declare that the research was conducted in the absence of any commercial or financial relationships that could be construed as a potential conflict of interest.

## Publisher’s Note

All claims expressed in this article are solely those of the authors and do not necessarily represent those of their affiliated organizations, or those of the publisher, the editors and the reviewers. Any product that may be evaluated in this article, or claim that may be made by its manufacturer, is not guaranteed or endorsed by the publisher.
